# Parents’ Working Conditions in the Early COVID-19 Pandemic and Children’s Health-Related Quality of Life: The Ciao Corona Study

**DOI:** 10.3389/ijph.2022.1605036

**Published:** 2022-08-24

**Authors:** Nevesthika Muralitharan, Gabriela P. Peralta, Sarah R. Haile, Thomas Radtke, Agne Ulyte, Milo A. Puhan, Susi Kriemler

**Affiliations:** ^1^ Department of Epidemiology, Epidemiology, Biostatistics and Prevention Institute, University of Zurich, Zürich, Switzerland; ^2^ Health Ethics and Policy Lab, Department of Health Sciences and Technology, ETH Zürich, Zürich, Switzerland

**Keywords:** children, health-related quality of life, COVID-19, parents, working conditions

## Abstract

**Objective:** To assess the associations between parents’ working conditions during the lockdown period (March-May 2020) and children’s health-related quality of life (HRQOL) over the first year of the COVID-19 pandemic in Zurich, Switzerland.

**Methods:** We included 2211 children (6–16 years) and their parents from the prospective study Ciao Corona. Parents reported their employment status and working conditions during the lockdown. Children’s HRQOL was assessed in June-July 2020, January and March 2021 using the parents-report of the KINDL®. We used mixed models to assess the associations between parents’ working conditions and children’s HRQOL at the three time points.

**Results:** Children from families in which at least one parent changed their working conditions during the lockdown showed lower HRQOL in June-July 2020, than children from families in which neither parent experienced changes. Children from families in which at least one parent had to work remotely continued to show lower HRQOL in January and March 2021.

**Conclusion:** Changes in parents’ working conditions during lockdown were negatively associated with children’s HRQOL. Public health policies aiming to support families susceptible to adverse changes are needed.

## Introduction

The measures put in place to mitigate the spread of the COVID-19 pandemic led to significant changes in adults’ and children’s daily life. The Swiss government imposed a strict lockdown between March and May 2020, which included suspension of classroom teaching and closure of care institutions [1]. During that period, most adults were forced to work remotely or to stop working, and only essential workers such as health workers were allowed to work on site. In June 2020, primary and secondary schools reopened, though with strict preventive measures in place. Due to the alarming increase of SARS-CoV-2 infections in autumn and winter 2020, the requirement to work from home was reinstated and remained in place until early 2021 while schools remained open [1].

Researchers around the globe have expressed their concerns about the potential deleterious effects of COVID-19 related restrictive measures and social constraints on children’s health and health-related quality of life (HRQOL) [2–4]. A systematic review assessing the impact of the pandemic on children’s and adolescents’ HRQOL concluded that HRQOL decreased during the first year of the pandemic [5]. Also, a Swiss study showed that HRQOL scores in children were particularly low during critical periods of the pandemic when sudden surges of infections happened [6]. Along with lower HRQOL, previous studies also reported an increase of mental health problems in children and adolescents [7, 8], particularly in those with low socioeconomic status, migration background and limited living space [7]. In addition, it has been suggested that the parental burden caused by the pandemic or by changes in their working conditions is shared with their children, contributing to lower HRQOL [9–12]. In line with this, a study in Germany showed that children whose parents were unemployed during the pandemic reported lower HRQOL than children with employed parents [9]. However, studies assessing the associations between changes of parents’ working conditions during the pandemic and children’s HRQOL are still scarce. Most of previous research focused only on parents’ employment status [7, 9] and to date only a cross-sectional study in Turkey assessed changes in parents’ working conditions during the lockdown in relation to their children’s HRQOL [13]. This study found that children whose parents’ were forced to stop working had the lowest HRQOL scores, followed by children whose parents had to work remotely [13]. However, this study included a single measure of children’s HRQOL and it was assessed in the early phase of the pandemic. Given the limitations of previous research, longitudinal studies that include data on parents’ working conditions and repeated measures of children’s HRQOL during the COVID-19 pandemic are needed to better understand this association.

The aim of this study was to assess the associations between parents’ working conditions during the lockdown period (i.e., 16 March to 11 May 2020) and children’s HRQOL over the first year of the pandemic in Zurich, Switzerland.

## Methods

### Study Sample and Design

We used data from Ciao Corona, a population-based prospective longitudinal cohort study investigating SARS-CoV-2 seroprevalence and clustering of cases among school-aged children in the canton of Zurich, Switzerland. The detailed protocol and study design can be found elsewhere [14]. Briefly, primary schools were randomly selected from the list of all schools in the canton of Zurich, stratified by district, and matched with the geographically closest secondary school. Main exclusion criteria for schools were small school size (<40 children in a selected school level), and for participants suspected or confirmed infection with SARS-CoV-2 during the testing day, precluding their attendance of the school on that day. Of 156 schools invited to participate, 55 schools agreed. Out of the children and adolescents invited in these schools, 2,585 were enrolled from June 16 to 9 July 2020, with an approximate participation rate of 50% [15, 16].

After enrolment parents were invited to fill out a baseline questionnaire in June-July 2020 and two follow-up questionnaires in January and March 2021, including questions on sociodemographic and health related information of the participant child or adolescent and their family. In the present study, we included families who had filled out information on mother’s or father’s employment status and their working conditions during the lockdown period, as well as on children’s HRQOL at baseline (Supplementary Figure S1).

### Measures

#### Employment Status and Parents’ Working Conditions

We assessed mother’s and father’s employment status before the pandemic (i.e., before March 2020) and working conditions during the lockdown period (i.e., between 16 March–11 May 2020) in the baseline questionnaire. If parents reported to be employed before the pandemic, they were asked whether their working conditions changed during the lockdown period. Those who reported a change were asked to choose one of the following options: “working remotely,” “temporarily stopped working or reduced workload,” “sick leave related or not related to the pandemic,” “unemployed as a result of the pandemic.” We then grouped the last three categories as “reduced or loss of income.” To assess the family situation, we combined mother and father information in a single variable with four categories: “both regular working conditions,” “at least one working remotely,” “at least one reduced or loss of income,” and “at least one unemployed already before lockdown.” For each possible combination, the most adverse situation defined the category of the combined variable (e.g., if one parent had to work remotely and the other had a reduction or loss of income, the family was assigned to the ‘at least one reduced or loss of income’ category). We used this combined variable as main exposure in the analysis. The specific questions and more details on the derivation of the combined variable are provided in the supplement.

#### Health-Related Quality of Life

We assessed children’s HRQOL with reference to the previous week using the German version of the parents’ report of the KINDL® at baseline and in the two follow-ups. KINDL® is a validated questionnaire for children aged 3–17 years that consists of 24 Likert-scaled items associated with six dimensions: physical well-being, emotional well-being, self-esteem, family, friends and school [17]. Normalized scores (ranging from 0 to 100) were calculated for each dimension and a normalized total score by combining all six dimensions. A higher total score represents a higher HRQOL [17]. We used the total score as the main outcome variable, and the scores for the subscales for sensitivity analyses.

#### Covariates

Sociodemographic and other health data were also collected in the baseline questionnaire. These included child’s sex, age and presence of any chronic conditions. We categorized age in two groups: 6–12 years and 13–16 years, which corresponds to primary and secondary school, respectively. We also collected data on parents’ nationality (at least one Swiss or both non-Swiss), educational attainment (high: at least one parent with a university degree, including universities of applied sciences; low/medium: both up to apprenticeship or professional school), mother’s and father’s age and on the number of persons living in the household, which was categorized as “≤3,” “4,” and “≥5.”

### Statistical Analysis

To avoid bias due to missing data, we implemented multiple imputations by chained equations for missing values for the exposure, outcome and covariates generating 25 complete datasets. Distributions among imputed values closely matched the observed values (Supplementary Table S2).

Descriptive statistics are reported as count (%) or mean with standard deviation (SD) as appropriate. Using linear mixed models, we assessed the associations between parents’ working conditions during the lockdown period and total KINDL score at the three time points (June-July 2020, January 2021, and March 2021), with school as random intercept. We built separate models for each time point, which were adjusted for child’s sex, age, presence of any chronic condition, parents’ nationality and educational attainment, mother’s age, father’s age and household size. As sensitivity analyses, we modeled the scores of the KINDL subscales and stratified the models by age group and parental education level. We also repeated the models using the complete case dataset (i.e., without imputation). The normality assumption for the adjusted models was confirmed by assessing the normal distribution of the residuals.

All statistical analyses were performed in R (version 4.0.3) [18] and the *lme4* package was used for the linear mixed models [19].

## Results

### Characteristics of the Study Sample

We included 2,211 school-aged children and their parents in the present analysis. The main characteristics of the study sample are presented in Table 1. Children’s mean age was 11.4 (SD: 2.5) years at baseline and most of them had at least one parent of Swiss origin (83%) and with a high education level (73%). Overall, 81% of mothers and 95% of fathers reported to be employed before the pandemic. Compared to the study sample, children and parents excluded from the analysis due to missing data were more likely to be non-Swiss and reported low/medium educational attainment (Supplementary Table S3).

**TABLE 1:** Baseline characteristics of the study population (Ciao Corona, Zürich, Switzerland, 2020–2021).

	Children and adolescents N = 2,211^a^
Age	11.4 (2.5)
Age-groups in children	
6–12 yrs.	1,451/2,211 (66%)
13–16 yrs.	760/2,211 (34%)
Sex	
Male	1,072/2,211 (48%)
Female	1,134/2,211 (51%)
Other	5/2,211 (0.2%)
Presence of chronic condition	
No	1,576/2,190 (72%)
Yes	614/2,190 (28%)
Age of mother yrs.	43.8 (5.0)
Age of father yrs.	46.7 (6.0)
Nationality of parents	
Swiss	1,826/2,201 (83%)
Other	375/2,201 (17%)
Parental education	
At least one high	1,563/2,138 (73%)
Both low/medium	575/2,138 (27%)
Employment status—Mother	
Employed	1,776/2,201 (81%)
Unemployed	425/2,201 (19%)
Employment status—Father	
Employed	2,074/2,174 (95%)
Unemployed	100/2,174 (4.6%)
Number of persons living in the household	
≤3	1,151/2,210 (52%)
4	352/2,210 (16%)
≥5	707/2,210 (32%)

Abbreviations: yrs., years.

a Values are means (standard deviation); n/N (%).

The mean total KINDL score was 77.9 (SD: 10) in June-July 2020, 76.3 (10.6) in January 2021 and 76.5 (10.9) in March 2021. Children aged 13–16 years reported lower scores than younger children (Table 2, Supplementary Table S4).

**TABLE 2:** Total KINDL scores over time stratified by age groups (Ciao Corona, Zürich, Switzerland, 2020–2021).

	June-July 2020	January 2021	March 2021
Overall, N = 2,211	77.9 (10.0)	76.3 (10.6)	76.5 (10.9)
Age group			
6–12 yrs, N = 1,451	79.9 (9.3)	78.3 (9.8)	78.5 (10.0)
13–16 yrs, N = 760	74.2 (10.3)	72.0 (10.9)	72.1 (11.5)

Abbreviations: yrs., years. Values are means (standard deviation).

### Parents’ Working Conditions

**FIGURE 1:**
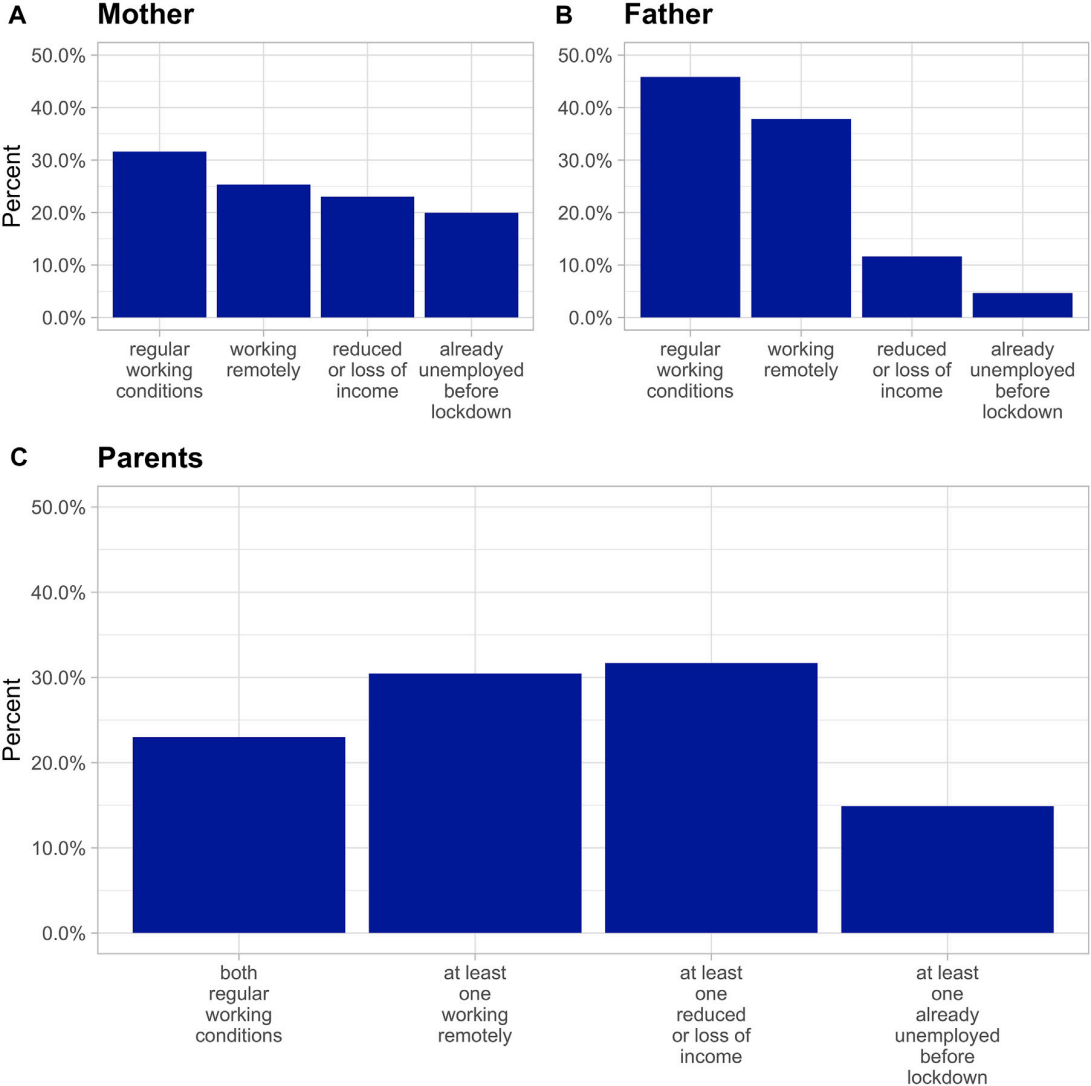
Working conditions for mothers **(A)**, fathers **(B)** and both parents **(C)** during the lockdown period (16th March to 11th May 2020). Parents of the participants who were employed before the start of the pandemic reported changes in their working conditions during the lockdown (“regular working conditions,” “working remotely,” “reduced or loss of income”). Some parents were already unemployed before the pandemic (“already unemployed before lockdown”). The variable for parents was derived by combining father’s and mother’s working conditions during the lockdown. (Ciao Corona, Zürich, Switzerland, 2020–2021).

Figure 1 shows the change of working conditions for mothers and fathers during the lockdown period. In general, most mothers and fathers experienced regular working conditions during the lockdown, followed by mothers and fathers who worked remotely. A higher proportion of mothers than fathers reported to be unemployed already before the pandemic (20% and 5%, respectively). In addition, a higher proportion of mothers who were employed before the pandemic faced reductions or loss of income during lockdown compared to fathers (23% and 12%, respectively).

Figure 1C shows the combined variable for changes in working conditions for both parents combined. In 23% of the families, both parents experienced regular working conditions during the lockdown period while in 30% at least one parent had to work remotely. Thirty-two percent of the families reported that at least one parent experienced a reduction or loss of income during the lockdown.

### Associations Between Parents’ Working Conditions and HRQOL

Table 3 shows the adjusted associations between parents’ working conditions during the lockdown period and the repeated total KINDL scores covering a 9-month period (June-July 2020, January and March 2021). Compared to children from families in which both parents experienced regular working conditions, children from families in which at least one parent had reduced or loss of income had the lowest total KINDL score in June-July 2020 (regression coefficient = −2.65 [95% confidence interval −3.81 to −1.48]), followed by children from families in which at least one parent had to work remotely (−1.91 [−3.07 to −0.74]), and children from families in which at least one parent was already unemployed before the lockdown (−1.43[−2.85 to −0.02]).

**TABLE 3:** Adjusted associations between changes in parents’ working conditions during the lockdown period and children’s total KINDL scores over time (Ciao Corona, Zürich, Switzerland, 2020–2021).

Predictors	KINDL total score June-July 2020		KINDL total score January 2021		KINDL total score March 2021	
	Coef. [95% CI]	*p*	Coef. [95% CI]	*p*	Coef. [95% CI]	*p*
Both regular working conditions	References		References		References	
At least one working remotely	−1.91 [−3.07 to −0.74]	0.001	−1.64 [−2.98 to −0.3]	0.017	−2.74 [−4.11 to −1.37]	<0.001
At least one reduced or loss of income	−2.65 [−3.81 to −1.48]	<0.001	−1.59 [−2.87 to −0.3]	0.016	−2.65 [−4.14 to −1.17]	<0.001
At least one already unemployed before lockdown	−1.43 [−2.85 to −0.02]	0.047	−0.2 [−1.84 to 1.44]	0.813	−0.82 [−2.53 to 0.89]	0.346

Abbreviations: Coef., regression coefficient; 95% CI, 95% confidence interval; p, *p*-value.

Models were adjusted for children’s age and sex, presence of chronic condition, mother’s age, father’s age, parental nationality and educational attainment, and household size.

Compared to children from families in which neither parent experienced a change, children from families in which at least one parent had to work remotely and in which at least one parent had reduced or loss of income during the lockdown continued to have lower total KINDL scores in January and March 2021. The direction of the associations remained stable in all sensitivity analyses (Supplementary Tables S5–S12). However, the category of ‘at least one parent was already unemployed’ did not appear to be associated with most of the individual subscales and the regression estimates were less precise for the analysis stratified by child’s age group and parents’ education level. Overall, the effect estimates of the complete case analysis did not differ from the model using imputed data (Supplementary Table S13).

## Discussion

In this population-based prospective longitudinal cohort study we found that changes in parents’ working conditions during the lockdown period (March–May 2020) were associated with children’s HRQOL at different time points during the first year of the COVID-19 pandemic in Zurich, Switzerland. Children from families in which at least one parent had to work remotely, had reduced or loss of income, or was already unemployed before lockdown had lower HRQOL in June-July 2020 compared to children from families in which both parents maintained regular working conditions. In addition, we observed that children from families in which at least one parent had to work remotely or had reduced or loss of income during the lockdown continued to have lower HRQOL in January and March 2021.

Our findings are in line with previous research showing that parents’ socioeconomic status and working conditions are predictors of children’s wellbeing and quality of life [13]. We found that children from families in which at least one parent had reduced or loss of income during the lockdown had the lowest HRQOL in June-July 2020 and continued to show lower HRQOL in January and March 2021. This is in line with evidence that children from families with financial hardship are likely to report lower HRQOL and higher prevalence of mental health problems [5, 7, 20, 21]. This association can be explained in part by the fact that children with lower socioeconomic status are likely to have fewer material resources, less social support and usually share a small living area [22, 23]. Especially in vulnerable families where parents feel an uncertainty and fear about the future, children become aware of their parents’ emotional states [24]. In fact, a previous study showed that adolescents who perceived the economic status of their family as low, were prone to depressive symptoms during the lockdown [11]. Finally, it is also possible that job loss, financial stress and reduced access to social support systems during the lockdown period increased the risk of domestic violence and child abuse [22], contributing to the decrease in children’s HRQOL.

We also found that children from families in which at least one parent had to work remotely during the lockdown had lower HRQOL at all time points (i.e., June-July 2020, January, and March 2021), which is consistent with a previous cross-sectional study using the same HRQOL measurement tool [13]. These associations might be explained by the constant presence at home of all family members, which is likely to increase personal conflicts. Indeed, previous studies showed that children quarreled more with parents during strict home confinement periods [24], particularly in families of low socioeconomic status [5]. In addition, children had to spend more time with their parents at home and their limited opportunities for social interactions outside home may have contributed to make them feel less autonomous and more lonely [3, 11]. Consequently, parents may have taken on a heavy burden during the pandemic as well. Parents that were dependent on care institutions suddenly faced additional childcare at home along with home office, which could be overwhelming. Not surprisingly, a recent German study found that parents working from home were also less satisfied with the care of their children [25], which could adversely affect the relationship among them. The lack of stable and supportive relationships and the permanent presence of both parents at home enabling conflict or disabling privacy could also have detrimental effects on the cognitive stage of development, especially for adolescents [26]. Indeed, we found that parents’ working conditions during the lockdown significantly affected the *family* KINDL subscale.

Importantly, we observed that children from families in which at least one parent had a change in working conditions during the lockdown continued to have low HRQOL in January and March 2021. In contrast, this was not the case for families in which at least one parent was already unemployed before the pandemic. It is possible that parents who had to work remotely during lockdown had to continue to do so for a longer period of time or even until early 2021. Although the lockdown was lifted by mid-May 2020, the recommendation to work from home for most office jobs, and especially in large companies, was in place until early 2021. Due to the increase of infections in autumn and winter of 2020 there was also a period of mandated home office in those months [27]. Additionally, individuals from certain job sectors, for example those working in restaurants or non-essential shops, had to stop working or had to reduce their working hours again during the second wave. Therefore, it is not surprising that children with parents who were likely to maintain worse working conditions after the initial lockdown continued to show low HRQOL scores in January and March 2021. Interestingly, children from families in which one parent was already unemployed before the pandemic did not show low HRQOL scores after June-July 2020. In line with this finding, a German study reported that unemployed parents, especially homemakers, showed the highest satisfaction with their childcare at home after the lockdown period [25] compared to employed parents. Despite the loss of work before the pandemic, the financial security may have been at least partly guaranteed due to unemployment benefits. In fact, a previous report showed that families who lost their jobs during the pandemic but maintained their household income (e.g., through compensation or through their partner’s job) reported better family interactions as well [28].

Our findings have important implications for public health and future research. Our results demonstrated that changes in parents’ working conditions due to COVID-19 restrictions can be considered as risk factors for decreased HRQOL in children, particularly in those families in which at least one parent experienced remote working or a reduction or loss of income. Given the already high number of risk factors for impaired HRQOL in the COVID-19 pandemic, these finding highlight the urgent need for policymakers to support not only children, but also families susceptible to adverse working conditions. Schools could implement measures to unburden parents who have to work at home and to support children to maintain their social support. In addition, future research that includes repeated measures of children’s HRQOL as well as of parents’ working conditions during and beyond the pandemic restrictions is needed to better understand the associations between these two factors. Future studies should make a special effort to include families with a low socioeconomic status and pay special attention to the barriers that prevent them from participating in epidemiological studies, such as language barriers or low health literacy. Families with a low socioeconomic status or migrant background are likely to be more affected by unfavorable changes in working conditions and, therefore children from these families may face a higher burden for their mental health and quality of life.

### Strengths and Limitations

Important strengths of this study are the large sample size, its population-based sampling and the longitudinal design, as well as the availability of repeated measures of HRQOL over the first year of the COVID-19 pandemic. In addition, we collected information on employment status and working conditions both for mothers and fathers, which allowed us to create a combined variable that represented changes on the family level.

However, we need to acknowledge some limitations. Children’s HRQOL was reported by parents therefore some bias is possible for these measures. Due to the lack of information about the minimal important difference in the KINDL questionnaire, we cannot weigh the meaningfulness of the reduction in HRQOL associated with adverse working conditions. Also, the fact that participants included in the study were more likely to be from families with Swiss nationality and a high education level than those excluded due to missing data, may not allow to generalize our findings to populations with more ethnic variability and lower socio-economic background. In addition, we had information about parents’ working conditions only during the lockdown period, and therefore, we could not assess long-term changes. Finally, although we account for several potential confounders when assessing the associations between parents’ working conditions and children’s HRQOL, some potential residual confounding is possible as we did not collect information on family income or size of the house.

### Conclusion

In summary, our study shows that changes in parents’ working conditions during the lockdown period were associated with children’s HRQOL over the first year of the COVID-19 pandemic. Children from families with more financial limitations (i.e., due to reduced or loss of income) and from families in which at least one parent had to work remotely were those who showed lower HRQOL from after the lockdown to January 2021. Public health policies aiming to reduce the burden of COVID-19 pandemic on children are urgently needed, and not only targeting children but also families susceptible to adverse working conditions.
