# Supplementary material for: Parents’ Working Conditions in the Early COVID-19 Pandemic and Children’s Health-Related Quality of Life: The Ciao Corona Study
Source: Int J Public Health. 2022 Aug 24;67:1605036. doi: 10.3389/ijph.2022.1605036 (PMC9448850; doi:10.3389/ijph.2022.1605036)
Supplement: Supplementary file 1 [file DataSheet1.pdf]

SUPPLEMENTARY APPENDIX TO:

Parents' working conditions in the early COVID-19 pandemic and children's health-related quality of life: the Ciao Corona study

Table of contents

Supplementary methods

Question formulation for assessing employment status and changes in mothers' and fathers' working conditions during the lockdown.

Supplementary results

Figure S1. Flowchart of the study population

Table S1. Derivation of parents' variable for changes in working conditions

Table S2. Distribution of imputed characteristics in the imputation datasets and in the observed data.

Table S3. Characteristics of participants included and excluded from the study.

Table S4. KINDL scores over time stratified by age groups.

Table S5. Adjusted associations between changes in parents' working conditions during the lockdown period and children's physical well-being score over time.

Table S6. Adjusted associations between changes in parents' working conditions during the lockdown period and children's emotional well-being score over time.

Table S7. Adjusted associations between changes in parents' working conditions during the lockdown period and children's self-esteem score over time.

Table S8. Adjusted associations between changes in parents' working conditions during the lockdown period and children's family score over time.

Table S9. Adjusted associations between changes in parents' working conditions during the lockdown period and children's friends score over time.

Table S10. Adjusted associations between changes in parents' working conditions during the lockdown period and children's school score over time.

Table S11. Adjusted associations between changes in parents' working conditions and children's total KINDL scores over time – Stratified by age groups.

Table S12. Adjusted associations between changes in parents' working conditions and children's total KINDL scores over time – Stratified by parental education level.

Table S13. Adjusted associations between changes in parents' working conditions during the lockdown period and children's total KINDL scores over time in the observed data

Figure S1. Flowchart of the study population (Ciao Corona, Switzerland, 2020-2021)

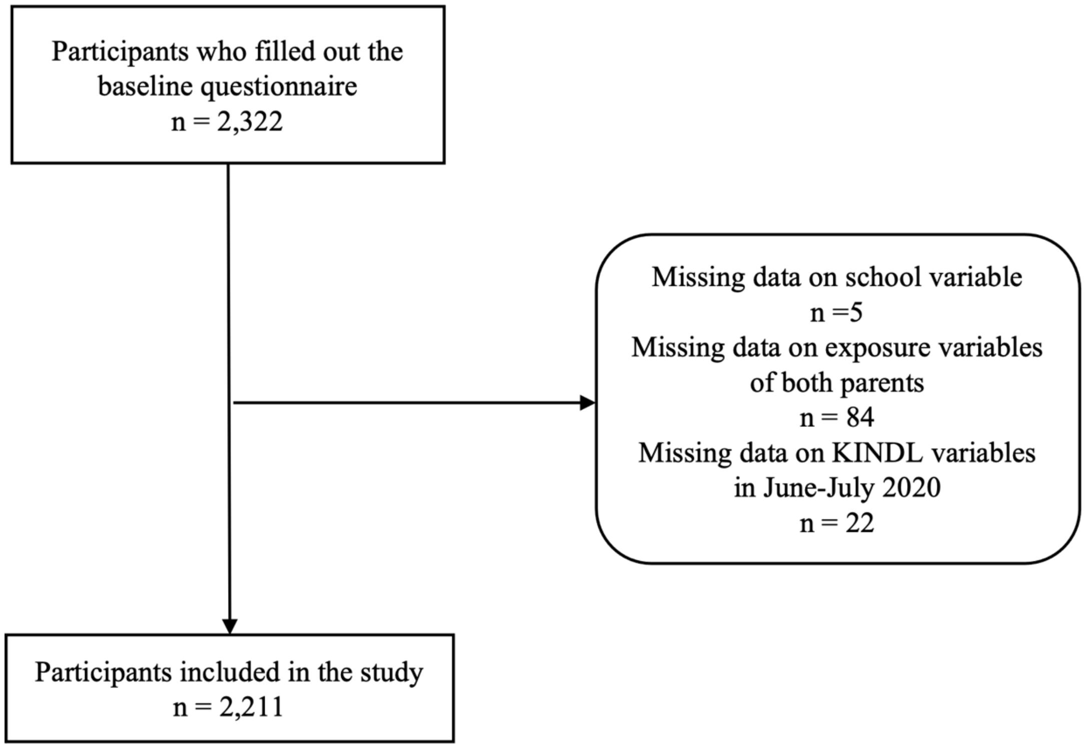

Question formulation (translated from German) for assessing employment status and mothers' and fathers' working conditions during the lockdown (Ciao Corona, Switzerland, 2020-2021)

Mother:

As a mother (partner of the father), are you employed or were you employed until the Corona crisis?

a. You are employed.
b. You are not employed

Did working conditions of the mother (partner of the father) change during the lockdown (period 16 March to 11 May 2020)?

a. Yes
b. No

If yes, please specify:

a. You stopped working or reduced your work temporarily
b. You worked more remotely
c. You were on a sick leave (with or without a relation to a corona infection)
d. You were unemployed as a result of the corona crisis
e. Other

Father:

As a father (partner of the mother), are you employed or were you employed until the Corona crisis?

a. You are employed.
b. You are not employed

Did working conditions of the father (partner of the mother) change during the lockdown (period 16 March to 11 May 2020)?

a. Yes
b. No

If yes, please specify:

a. You stopped working or reduced your work temporarily
b. You worked more remotely
c. You were on a sick leave (with or without a relation to a corona infection)
d. You were unemployed as a result of the corona crisis
e. Other

Table S1. Derivation of the combined variable for parents' working conditions during the lockdown period (Ciao Corona, Switzerland, 2020-2021)

		**Father**			
		Regular working conditions	Working remotely	Reduced or loss of income	Already unemployed before lockdown
**Mother**	Regular working conditions	a	b	c	d
	Working remotely	b	b	c	b
	Reduced or loss of income	c	c	c	c
	Already unemployed before lockdown	d	b	c	d

New categories:

a) both regular working conditions
b) at least one working remotely
c) at least one reduced or loss of income
d) at least one already unemployed before lockdown

Table S2. Distribution of children's and parents' characteristics in the observed and the imputed datasets (Ciao Corona, Switzerland, 2020-2021)

Variables	%missing	Observed dataset	Imputed datasets
**Age**	0	11.4 (2.5)	11.4 (2.5)
**Sex,** %	0		
Male		48	48
Female		51	51
Other		0.2	0.2
**Presence of chronic condition**			
No		72	72
Yes		28	28
**Age of mother yrs.**	11	43.8 (5.0)	43.9 (5.0)
**Age of father yrs.**	17	46.7 (6.0)	46.8 (6.1)
**Nationality of parents**, %	0.5		
Swiss		83	83
Other		17	17
**Parental education**, %	3.3		
At least one high		73	72
Both low/medium		27	28
**Changes in working conditions for parents**, %	12		
Both regular working conditions		23	23
At least one working remotely		30	30
At least one reduced or loss of income		32	32
At least one already unemployed before lockdown		15	15
**Number of persons in the same household**, %	<0.1		
<=3		52	52
4		16	16
>=5		32	32
**Total KINDL score in June 2020**	0.4	77.9 (10.0)	77.9 (10.0)
**Total KINDL score in January 2021**	22	76.3 (10.6)	75.9 (10.6)
**Total KINDL score in March 2021**	25	76.5 (10.9)	76.0 (10.9)

Abbreviations: yrs.: years
[a] Mean (standard deviation); %

Table S3. Characteristics of participants included and excluded from the study (Ciao Corona, Switzerland, 2020-2021)

**Variables**	**Included**<br>N = 2,211	**Excluded**<br>N = 111	**P-value** [b]
**Age**	11.4 (2.5)	12.3 (2.5)	<0.001
**Age-groups in children**			<0.001
6-12 yrs.	1,451 / 2,211 (66%)	51 / 110 (46%)	
13-16 yrs.	760 / 2,211 (34%)	59 / 110 (54%)	
**Gender**			0.041
Male	1,072 / 2,211 (48%)	49 / 110 (45%)	
Female	1,134 / 2,211 (51%)	59 / 110 (54%)	
Other	5 / 2,211 (0.2%)	2 / 110 (1.8%)	
**Presence of chronic condition**			0.800
no	1,576 / 2,190 (72%)	78 / 107 (73%)	
yes	614 / 2,190 (28%)	29 / 107 (27%)	
**Age of mother yrs.**	43.8 (5.0)	41.7 (5.0)	0.006
**Age of father yrs.**	46.7 (6.0)	45.8 (6.2)	0.500
**Nationality of parents**			<0.001
Swiss	1,826 / 2,201 (83%)	35 / 67 (52%)	
Other	375 / 2,201 (17%)	32 / 67 (48%)	
**Parental education**			0.010
At least one high	1,563 / 2,138 (73%)	29 / 51 (57%)	
At least one low/medium	575 / 2,138 (27%)	22 / 51 (43%)	
**Number of persons in the same household**			0.300
<=3	1,151 / 2,210 (52%)	53 / 110 (48%)	
4	352 / 2,210 (16%)	24 / 110 (22%)	
>=5	707 / 2,210 (32%)	33 / 110 (30%)	

Values are means (standard deviation) or n / N (%)

[b] The p-values correspond to Wilcoxon rank sum test, Pearson's Chi-squared test or to Fisher’s exact test.

Table S4. Total and subscale KINDL scores over time stratified by age groups (Ciao Corona, Switzerland, 2020-2021)

**Age groups**	**SUBSCALES**						**TOTAL SCORE**
	**Physical well-being**	**Emotional well-being**	**Self-esteem**	**Family**	**Friends**	**School**	
			**JUNE-JULY 2020:**				
**Overall**, N = 2,211	79.9 (15.1)	80.8 (13.6)	72.4 (15.1)	79.3 (13.6)	77.9 (14.0)	77.4 (16.2)	77.9 (10.0)
**6-12 yrs.**, N = 1,451	81.7 (14.6)	82.0 (12.9)	74.9 (14.0)	79.9 (13.0)	78.7 (13.6)	82.1 (14.4)	79.9 (9.3)
**13-16 yrs.**, N = 760	76.4 (15.5)	78.6 (14.5)	67.5 (15.9)	78.1 (14.6)	76.2 (14.7)	68.4 (15.4)	74.2 (10.3)
			**JANUARY 2021:**				
**Overall**, N = 2,211	77.7 (14.9)	79.2 (14.2)	71.6 (14.2)	78.6 (13.6)	76.4 (13.3)	74.2 (16.8)	76.3 (10.6)
**6-12 yrs.**, N = 1,451	80.0 (13.9)	80.9 (13.2)	73.7 (13.4)	79.3 (12.9)	77.3 (13.0)	78.5 (15.4)	78.3 (9.8)
**13-16 yrs.**, N = 760	72.7 (15.7)	75.5 (15.5)	67.2 (15.0)	77.2 (14.9)	74.6 (13.8)	65.0 (15.9)	72.0 (10.9)
			**MARCH 2021:**				
**Overall**, N = 2,211	77.4 (15.3)	79.3 (14.7)	71.8 (14.1)	78.3 (13.8)	77.4 (13.5)	74.7 (16.2)	76.5 (10.9)
**6-12 yrs.**, N = 1,451	79.6 (14.5)	80.9 (13.6)	73.9 (13.4)	79.2 (12.9)	78.4 (13.2)	78.8 (14.8)	78.5 (10.0)
**13-16 yrs.**, N = 760	72.6 (15.9)	75.7 (16.4)	67.3 (14.6)	76.4 (15.5)	75.1 (13.7)	65.6 (15.4)	72.1 (11.5)

Abbreviations: yrs.: years. Values are means (standard deviation).

Table S5. Adjusted associations between changes in parents' working conditions during the lockdown period and children's physical well-being score over time (Ciao Corona, Switzerland, 2020-2021)

	Physical well-being score June-July 2020		Physical well-being score January 2021		Physical well-being score March 2021	
Predictors	*Coef. [95% CI]*	*p*	*Coef. [95% CI]*	*p*	*Coef. [95% CI]*	*p*
Both regular working conditions	Reference		Reference		Reference	
At least one working remotely	-3.51 [-5.33 to -1.7]	<0.001	-2.5 [-4.45 to -0.55]	0.012	-2.12 [-4.14 to -0.1]	0.04
At least one reduced or loss of income	-3.49 [-5.28 to -1.7]	<0.001	-2.95 [-4.79 to -1.11]	0.002	-2.00 [-4.08 to 0.08]	0.059
At least one already unemployed before lockdown	-1.79 [-3.97 to 0.39]	0.107	-1.50 [-4.01 to 1.02]	0.243	-0.66 [-3.07 to 1.76]	0.592

Abbreviations: Coef.: regression coefficient; 95% CI: 95% confidence interval; p: p-value
Models were adjusted for children's age and sex, presence of chronic condition, mother's age, father's age, parental nationality and educational attainment, and household size.

Table S6. Adjusted associations between changes in parents' working conditions during the lockdown period and children's emotional well-being score over time (Ciao Corona, Switzerland, 2020-2021)

	**Emotional well-being score June-July 2020**		**Emotional well-being score January 2021**		**Emotional well-being score March 2021**	
Predictors	*Coef. [95% CI]*	*p*	*Coef. [95% CI]*	*p*	*Coef. [95% CI]*	*p*
Both regular working conditions	Reference		Reference		Reference	
At least one working remotely	-1.25 [-2.9 to 0.4]	0.14	-2.46 [-4.39 to -0.54]	0.012	-3.62 [-5.64 to -1.61]	<0.001
At least one reduced or loss of income	-1.85 [-3.49 to -0.21]	0.03	-1.61 [-3.47 to 0.25]	0.09	-3.25 [-5.4 to -1.1]	0.003
At least one already unemployed before lockdown	-0.94 [-2.92 to 1.04]	0.35	-0.97 [-3.26 to 1.32]	0.405	-1.61 [-4.11 to 0.89]	0.205

Abbreviations: Coef.: regression coefficient; 95% CI: 95% confidence interval; p: p-value
Models were adjusted for children's age and sex, presence of chronic condition, mother's age, father's age, parental nationality and educational attainment, and household size.

Table S7. Adjusted associations between changes in parents' working conditions during the lockdown period and children's self-esteem score over time (Ciao Corona, Switzerland, 2020-2021)

	**Self-esteem score June-July 2020**		**Self-esteem score January 2021**		**Self-esteem score March 2021**	
Predictors	*Coef. [95% CI]*	*p*	*Coef. [95% CI]*	*p*	*Coef. [95% CI]*	*p*
Both regular working conditions	Reference		Reference		Reference	
At least one working remotely	-0.78 [-2.58 to 1.01]	0.394	-0.25 [-2.13 to 1.63]	0.8	-2.57 [-4.46 to -0.68]	0.008
At least one reduced or loss of income	-2.43 [-4.19 to -0.68]	0.006	-0.14 [-1.95 to 1.67]	0.88	-2.25 [-4.26 to -0.25]	0.028
At least one already unemployed before lockdown	-1.82 [-3.98 to 0.35]	0.1	-0.03 [-2.36 to 2.3]	0.98	-1.22 [-3.6 to 1.17]	0.315

Abbreviations: Coef.: regression coefficient; 95% CI: 95% confidence interval; p: p-value
Models were adjusted for children's age and sex, presence of chronic condition, mother's age, father's age, parental nationality and educational attainment, and household size.

Table S8. Adjusted associations between changes in parents' working conditions during the lockdown period and children's family score over time (Ciao Corona, Switzerland, 2020-2021)

	**Family score June-July 2020**		**Family score January 2021**		**Family score March 2021**	
Predictors	*Coef. [95% CI]*	*p*	*Coef. [95% CI]*	*p*	*Coef. [95% CI]*	*p*
Both regular working conditions	Reference		Reference		Reference	
At least one working remotely	-1.66 [-3.32 to 0]	0.05	-2.32 [-4.13 to -0.52]	0.012	-2.82 [-4.64 to -1.01]	0.002
At least one reduced or loss of income	-3.09 [-4.7 to -1.47]	<0.001	-1.74 [-3.48 to 0]	0.05	-2.74 [-4.59 to -0.9]	0.004
At least one already unemployed before lockdown	0.24 [-1.76 to 2.23]	0.82	-0.3 [-2.61 to 2.01]	0.798	0.4 [-1.73 to 2.52]	0.716

Abbreviations: Coef.: regression coefficient; 95% CI: 95% confidence interval; p: p-value
Models were adjusted for children's age and sex, presence of chronic condition, mother's age, father's age, parental nationality and educational attainment, and household size.

Table S9. Adjusted associations between changes in parents' working conditions during the lockdown period and children's friends score over time (Ciao Corona, Switzerland, 2020-2021)

	**Friends score June-July 2020**		**Friends score January 2021**		**Friends score March 2021**	
Predictors	*Coef. [95% CI]*	*p*	*Coef. [95% CI]*	*p*	*Coef. [95% CI]*	*p*
Both regular working conditions	Reference		Reference		Reference	
At least one working remotely	-2.57 [-4.28 to -0.85]	0.003	-1.26 [-3.08 to 0.56]	0.174	-2.66 [-4.45 to -0.86]	0.004
At least one reduced or loss of income	-3.18 [-4.87 to -1.49]	<0.001	-1.32 [-2.98 to 0.35]	0.122	-2.57 [-4.47 to -0.67]	0.008
At least one already unemployed before lockdown	-2.34 [-4.41 to -0.28]	0.026	1.01 [-1.09 to 3.11]	0.345	-0.56 [-2.88 to 1.76]	0.636

Abbreviations: Coef.: regression coefficient; 95% CI: 95% confidence interval; p: p-value
Models were adjusted for children's age and sex, presence of chronic condition, mother's age, father's age, parental nationality and educational attainment, and household size.

Table S10. Adjusted associations between changes in parents' working conditions during the lockdown period and children's school score over time (Ciao Corona, Switzerland, 2020-2021)

	**School score June-July 2020**		**School score January 2021**		**School score March 2021**	
Predictors	*Coef. [95% CI]*	*p*	*Coef. [95% CI]*	*p*	*Coef. [95% CI]*	*p*
Both regular working conditions	Reference		Reference		Reference	
At least one working remotely	-1.67 [-3.42 to 0.08]	0.061	-0.77 [-2.74 to 1.2]	0.445	-2.64 [-4.66 to -0.63]	0.01
At least one reduced or loss of income	-1.9 [-3.63 to -0.16]	0.032	-1.37 [-3.3 to 0.57]	0.167	-3.04 [-4.99 to -1.09]	0.002
At least one already unemployed before lockdown	-2.05 [-4.18 to 0.09]	0.06	0.98 [-1.46 to 3.42]	0.429	-1.26 [-3.83 to 1.31]	0.336

Abbreviations: Coef.: regression coefficient; 95% CI: 95% confidence interval; p: p-value
Models were adjusted for children's age and sex, presence of chronic condition, mother's age, father's age, parental nationality and educational attainment, and household size.

Table S11. Adjusted associations between changes in parents' working conditions and children's total KINDL scores over time and stratified by age groups (Ciao Corona, Switzerland, 2020-2021)

	Total KINDL score June-July 2020		Total KINDL score January 2021		Total KINDL score March 2021	
Predictors	*Coef.[95% CI]*	*p*	*Coef.[95% CI]*	*p*	*Coef.[95% CI]*	*p*
**6-12 YRS.**						
Both regular working conditions	Reference		Reference		Reference	
At least one working remotely	-0.82 [-2.93 to 1.3]	0.45	-0.6 [-3.02 to 1.82]	0.626	-2.26 [-4.63 to 0.1]	0.061
At least one reduced or loss of income	-2.23 [-4.44 to -0.03]	0.05	-2.79 [-5.21 to -0.37]	0.024	-3.96 [-6.41 to -1.5]	0.002
At least one already unemployed before lockdown	-1.97 [-4.61 to 0.67]	0.14	0.38 [-2.56 to 3.32]	0.799	-1.96 [-4.98 to 1.05]	0.201
**13-16 YRS.**						
Both regular working conditions	Reference		Reference		Reference	
At least one working remotely	-1.62 [-4.98 to 1.74]	0.344	0.74 [-3.12 to 4.6]	0.71	-2.11 [-5.88 to 1.66]	0.27
At least one reduced or loss of income	-0.67 [-3.61 to 2.27]	0.655	1.53 [-1.99 to 5.06]	0.39	-1.04 [-4.34 to 2.26]	0.53
At least one already unemployed before lockdown	-3.16 [-6.94 to 0.63]	0.102	0.86 [-3.38 to 5.1]	0.69	-0.86 [-5.2 to 3.49]	0.7

Abbreviations: Coef.: regression coefficient; 95% CI: 95% confidence interval; p: p-value
Models were adjusted for children's sex, presence of chronic condition, mother's age, father's age, parental nationality and educational attainment, and household size.

Table S12. Adjusted associations between changes in parents' working conditions and children's total KINDL scores over time – Stratified by parental education level (Ciao Corona, Switzerland, 2020-2021)

	**Total KINDL score June-July 2020**		**Total KINDL score January 2021**		**Total KINDL score March 2021**	
Predictors	*Coef.[95% CI]*	*p*	*Coef.[95% CI]*	*p*	*Coef.[95% CI]*	*p*
**Low/medium education level**						
Both regular working conditions	Reference		Reference		Reference	
At least one working remotely	-1.52 [-5.77 to 2.74]	0.484	-0.29 [-5.26 to 4.67]	0.91	-3.31 [-8.29 to 1.66]	0.19
At least one reduced or loss of income	-0.16 [-3.19 to 2.88]	0.919	0.52 [-3.03 to 4.08]	0.77	-3.97 [-7.54 to -0.4]	0.03
At least one already unemployed before lockdown	1.28 [-2.53 to 5.1]	0.508	3.29 [-1.49 to 8.06]	0.18	-1.32 [-6.22 to 3.58]	0.59
**High education level**						
Both regular working conditions	Reference		Reference		Reference	
At least one working remotely	-2.2 [-4.17 to -0.23]	0.03	-1.42 [-3.76 to 0.91]	0.23	-2.56 [-4.78 to -0.33]	0.024
At least one reduced or loss of income	-2.47 [-4.63 to -0.31]	0.03	-2.12 [-4.67 to 0.43]	0.1	-2.4 [-4.82 to 0.02]	0.052
At least one already unemployed before lockdown	-2.27 [-4.9 to 0.36]	0.09	0.61 [-2.31 to 3.52]	0.68	-1.32 [-6.22 to 3.58]	0.59

Abbreviations: Coef.: regression coefficient; 95% CI: 95% confidence interval; p: p-value
Models were adjusted for children's age and sex, presence of chronic condition, mother's age, father's age, parental nationality, and household size.

Table S13. Adjusted associations between changes in parents' working conditions during the lockdown period and children's total KINDL scores over time in the observed data (Ciao Corona, Switzerland, 2020-2021)

	**KINDL total score June-July 2020**		**KINDL total score January 2021**		**KINDL total score March 2021**	
Predictors	*Coef. [95% CI]*	*p*	*Coef. [95% CI]*	*p*	*Coef. [95% CI]*	*p*
Both regular working conditions	Reference		Reference		Reference	
At least one working remotely	-1.81 [-3.10 to -0.52]	0.006	-1.61 [-3.12 to -0.11]	0.036	-2.21 [-3.80 to -0.61]	0.007
At least one reduced or loss of income	-2.46 [-3.74 to -1.18]	<0.001	-1.16 [-2.67 to 0.35]	0.132	-1.63 [-3.24 to -0.01]	0.048
At least one already unemployed before lockdown	-0.44 [-2.02 to 1.14]	0.584	1.28 [-0.62 to 3.17]	0.186	1.03 [-0.98 to 3.03]	0.316

Abbreviations: Coef.: regression coefficient; 95% CI: 95% confidence interval; p: p-value
Models were adjusted for children's age and sex, presence of chronic condition, mother's age, father's age, parental nationality and educational attainment, and household size.
